# Dorsal and ventral striatal functional connectivity shifts play a potential role in internet gaming disorder

**DOI:** 10.1038/s42003-021-02395-5

**Published:** 2021-07-14

**Authors:** Guang-Heng Dong, Haohao Dong, Min Wang, Jialin Zhang, Weiran Zhou, Xiaoxia Du, Marc N. Potenza

**Affiliations:** 1 Center for Cognition and Brain Disorders, the Affiliated Hospital of Hangzhou Normal University, Hangzhou, P.R. China; 2 Institute of Psychological Research, Hangzhou Normal University, Hangzhou, P.R. China; 3 Zhejiang Key Laboratory for Research in Assessment of Cognitive Impairments, Hangzhou, Zhejiang Province, P.R. China; 4 Department of Psychology, Nanjing University, Nanjing, P.R. China; 5 School of Psychology, Beijing Normal University, Beijing, China; 6 School of Psychology, Shanghai University of Sport, Shanghai, China; 7 Department of Psychiatry and Child Study Center, Yale University School of Medicine, New Haven, CT USA; 8 Department of Neuroscience, Yale University, New Haven, CT USA; 9 Connecticut Mental Health Center, New Haven, CT USA

**Keywords:** Human behaviour, Social behaviour

## Abstract

Animal models suggest transitions from non-addictive to addictive behavioral engagement are associated with ventral-to-dorsal striatal shifts. However, few studies have examined such features in humans, especially in internet gaming disorder (IGD), a proposed behavioral addiction. We recruited 418 subjects (174 with IGD; 244 with recreational game use (RGU)). Resting-state fMRI data were collected and functional connectivity analyses were performed based on ventral and dorsal striatal seeds. Correlations and follow-up spectrum dynamic causal model (spDCM) analyses were performed to examine relationships between the ventral/dorsal striatum and middle frontal gyrus (MFG). Longitudinal data were also analysed to investigate changes over time. IGD relative to RGU subjects showed lower ventral-striatum-to-MFG (mostly involving supplementary motor area (SMA)) and higher dorsal-striatum-to-MFG functional connectivity. spDCM revealed that left dorsal-striatum-to-MFG connectivity was correlated with IGD severity. Longitudinal data within IGD and RGU groups found greater dorsal striatal connectivity with the MFG in IGD versus RGU subjects. These findings suggest similar ventral-to-dorsal striatal shifts may operate in IGD and traditional addictions.

## Abstract (web summary)

In order to shed light on the underlying neural mechanisms of internet gaming disorder (IGD), Dong et al collected longitudinal resting-state fMRI data from participants with IGD or those who partake in recreational game use. They demonstrated that, consistent with animal models of addiction, dorsal and ventral striatal functional connectivity shifts appeared to play a potential mechanistic role in IGD.

## Introduction

Altered frontal-striatal communication may relate to impaired control over motivations that promote engagement in and persistence of addictive behaviours^1,2^. The striatum, important in reward processing, has been implicated in transitions in addictions. The striatum contributes to associative learning, motor function, behavioural control and other functions that may contribute to engagement in addictive behaviours^3^.

The striatum may be divided into ventral and dorsal components, based on functional evidence, and different striatal subdivisions may have distinct roles^2,3^. The dorsal striatum (DS) may be particularly important for flexible versus habitual action selection and action, and the ventral striatum (VS) may be particularly important for learning values of stimuli^4–6^.

Anatomically, the striatum receives respectively both glutamatergic and dopaminergic projections from multiple cortical and midbrain regions^7^. The VS receives major projections from prefrontal, temporal and limbic lobe, mainly including orbitofrontal, medial prefrontal, anterior prefrontal and anterior cingulate cortex. The DS has been divided into a dorsomedial portion (caudate), receiving projections primarily from association cortex (mainly dorsal lateral prefrontal cortex), and a dorsolateral portion (putamen), receiving projections primarily from sensory and motor areas. The VS also receives dopaminergic inputs primarily (but not exclusively) from the ventral tegmental area, and the DS receives dopaminergic afferents from the substantia nigra^8^. This heterogeneous connection within the striatum has multiple contributions to addiction-related processes. Animal models and theories of substance and behavioural addictions have suggested that transitions from non-addictive to addictive engagement may involve shifts in involvement of ventral-to-dorsal striatal cortico-striato-thalamo-cortical circuits^1,2,9^.

Human neuroimaging studies of drug craving indicate that drug-related cues activate DS regions implicated in habitual behaviours and linked to measures of compulsivity in drug-using populations^10–12^. Therefore, DS neuroadaptations have been implicated in transitions between incentive-based and habit-based control of behaviours, and greater involvement of the DS is often observed when substance/drug intake is more addictive or compulsive^3,13^.

Shifts in VS-DS function have recently been implicated in humans with cannabis^2^ or tobacco^14^ use disorders and those with obesity^3^. Behavioural addictions such as gambling and gaming disorders do not involve substances linked to the development of maintenance of these disorders or their recoveries^15–17^. Nonetheless, similar circuitry has been proposed to underlie such transitions^9^. However, few studies have examined such proposed models directly.

Internet gaming disorder (IGD) has been included in the DSM-5 as a possible condition warranting additional research^18^. IGD is characterised by poorly controlled gaming that leads to impairment or psychological distress and persists despite negative consequences^19,20^. Classified as a behavioural addiction, IGD may involve impaired executive control^21–23^, enhanced craving to gaming cues, and disadvantageous decision-making^16,19,24^.

Although studies have investigated neurobiological correlates of IGD, a minority has used resting-state fMRI assessments that may permit evaluations of VS-to-DS shifts that are not influenced by tasks or experimental paradigms^21,25,26^. Resting-state functional connectivity facilitates assessment of functional alterations in the absence of contextual modulation^27,28^. This approach has provided insight into frontostriatal functional circuits in cannabis^29,30^ and tobacco^14^ use disorders. Here we investigate the degree to which resting-state functional connectivity relates to aspects of poor impulse control or other potentially related features in IGD, particularly as resting-state fMRI measures have been related to individual differences in BOLD signals during task performance^31–33^.

Many people play video games in a controlled manner and exhibit recreational game use (RGU)^16,20,34^. The current study included people with RGU as a control group, and by comparing IGD to RGU subjects, we sought to examine brain features associated with IGD and potential changes over time. Identification of such features could provide new insights into neurobiological mechanisms of IGD.

Altogether, while both VS and DS networks have been proposed to be relevant to stages of IGD and other addictions, including with respect to addiction severity, few studies have directly assessed these relationships, particularly over time. Here, we aimed to investigate VS and DS functional connectivity in IGD versus RGU subjects and examine individual differences therein with respect to IGD severity. To do so, we applied a seed-based approach to resting-state fMRI data to assess VS and DS functional connectivity. Resting-state fluctuations may relate to cognitive and emotional biases that may in part shape individual preferences; thus, striatal connectivity measures may have predictive validity in relation to IGD severity^3,32,35,36^. As previous studies of VS-DS transitions observed increased functional connectivity involving the DS as described above, we hypothesised that IGD versus RGU participants would show relatively increased functional connectivity in the DS, particularly with respect to cortical regions implicated in IGD. We also hypothesised that functional connectivity involving the DS would persist over time in people continuing to exhibit IGD. Thus, we hypothesised that increased DS functional connectivity would be associated with IGD severity, and these relationships would be observed longitudinally.

## Results

### Ventral and dorsal features with brain regions

**Fig. 1:**
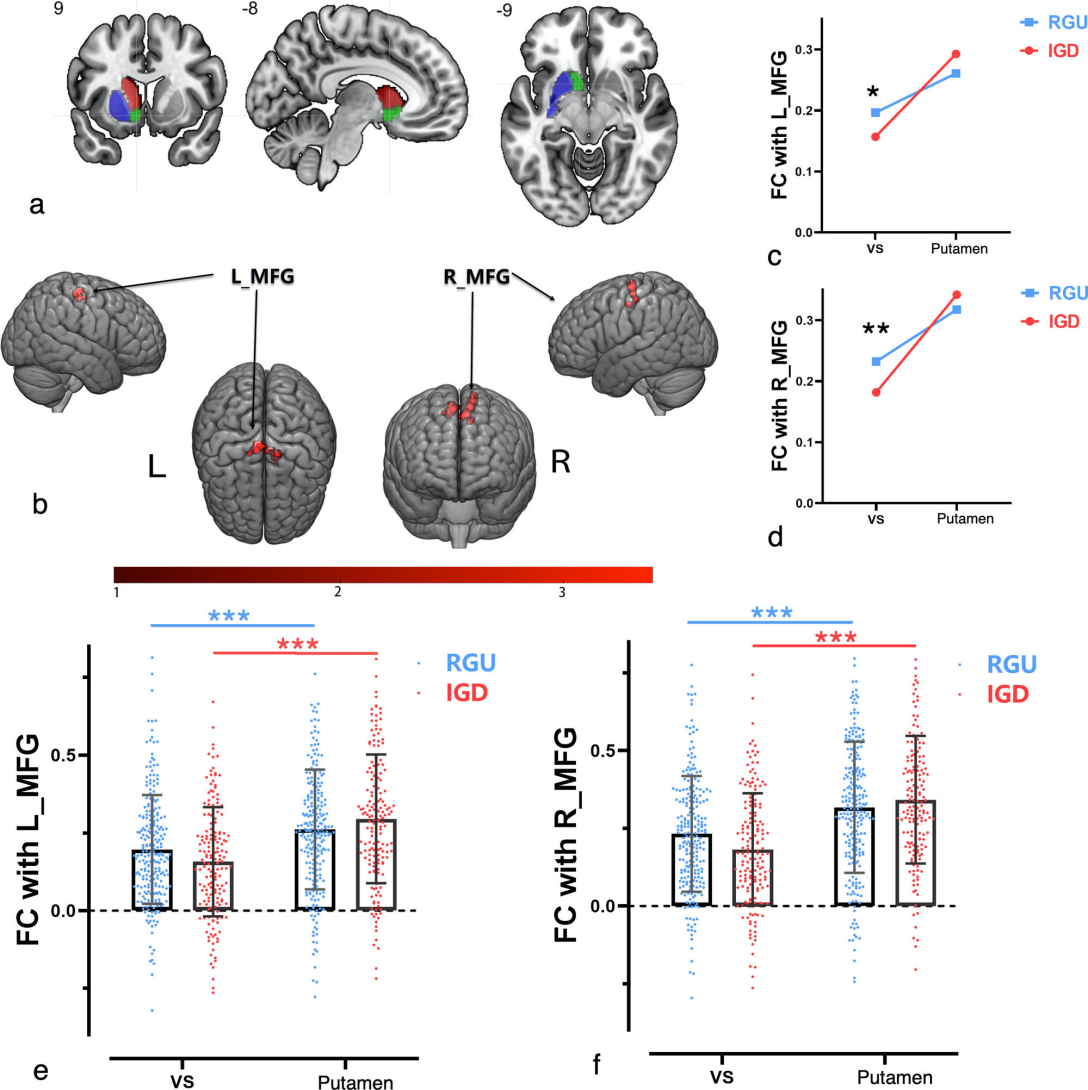
Interactions were found between ventral/dorsal striatum and the MFG. **a** ROIs selected for the ventral and dorsal striatum in the current study. Left hemispheric regions are shown. Green: NAcc; Red: Caudate; Blue: Putaman. **b** Brain regions that show functional connectivity interactions with the ventral/dorsal striatum seeds and diagnostic groups. **c**, **d** Plots demonstrating the interaction on functional connectivity strength between ventral striatum/putamen and left and right MFG in IGD and RGU subjects. **e**, **f** Strength of the functional connectivity between the VS/putamen and left or right MFG in IGD and RGU subjects. IGD: 174 subjects; RGU: 244 subjects. *p < 0.05; **p < 0.01.

When examining the left dorsal and ventral striatal region of interests (ROIs), significant interactions were observed in relation to MFG connectivity in IGD and RGU subjects (*F*(1, 416) = 19.293, *p* < 0.001, *η*^2^ = 0.044). Further post-hoc analyses showed that, in left hemisphere, IGD subjects showed lower VS-based functional connectivity (left MFG: *t*(416) = −2.278, *p* = 0.023, Cohens’*d* = 0.223) than RGU subjects, but no difference in the right hemisphere and putamen-based functional connectivity (right MFG: *t*(416) = 1.171, *p* = 0.242, Cohens’*d* = 0.115). IGD (relative to RGU) subjects showed statistically similar putamen-based functional connectivity (left MFG: *t*_(416)_ = 1.691, *p* = 0.092, Cohens’*d* = 0.166) and (right MFG: *t*(416) = 1.171, *p* = 0.242, Cohens’*d* = 0.115). Significant increases from VS to DS were observed in both hemispheres in RGU and IGD separately (Fig. 1 and Table 1).

**Table 1:** Results that show functional connectivity interactions between the VS and DS in the current study.

ROI	Cluster	Peak MNI coordinates			Peak intensity	Cluster size	Region	AAL
		*X*	*Y*	*Z*				
Left, Putamen- Accumbens	1	12	−15	63	4.58	55	R Medial Frontal Gyrus	Supp_Motor_Area_R
	2	−9	−24	57	4.3246	37	L Medial Frontal Gyrus	Supp_Motor_Area_L

All images are thresholded at *p* < 0.05, TFCE-corrected; Iterations = 5000.

*MNI* Montreal Neurological Institute, *AAL* anatomical automatic labelling, *TFCE* threshold-free clustering enhancement.

### Correlation with DSM-5 scores (Spearman Rho)

**Fig. 2:**
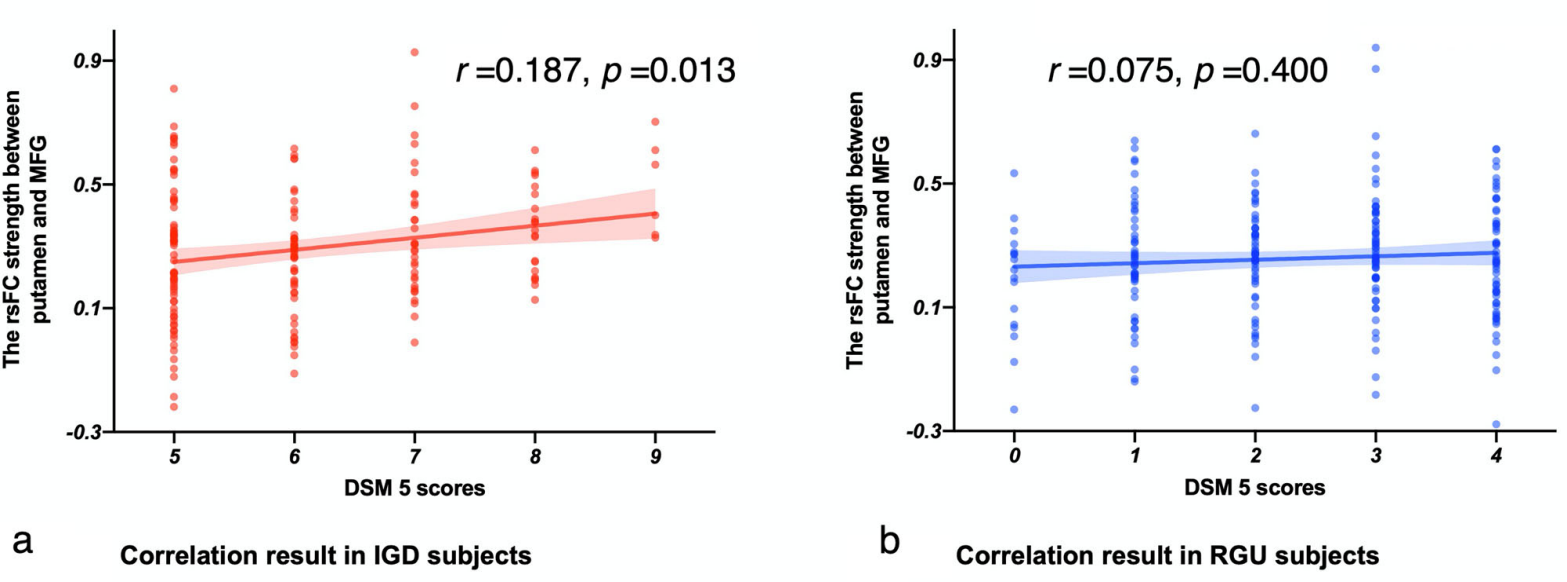
Correlations between IGD severity (DSM-5 score) and functional connectivity strength between left putamen (DS) and MFG. **a** The correlation results in IGD subjects. **b** The correlation results in RGU subjects. IGD: 174 subjects; RGU: 244 subjects.

A significant correlation was observed between left putamen-MFG communication and the DSM-5 scores in IGD subjects *(ρ* = 0.187, *p* = 0.013). No correlation was observed in RGU subjects *(ρ* = 0.056, *p* = 0.400) (Fig. 2). No correlations were observed in the VS-MFG functional connectivity with DSM-5 scores *(ρ* = −0.040, *p* = 0.439). As three correlation analyses were conducted here, the Bonferroni-corrected threshold for a significant *p* value would be *p* < 0.0167 (0.05/3).

### spDCM results

**Fig. 3:**
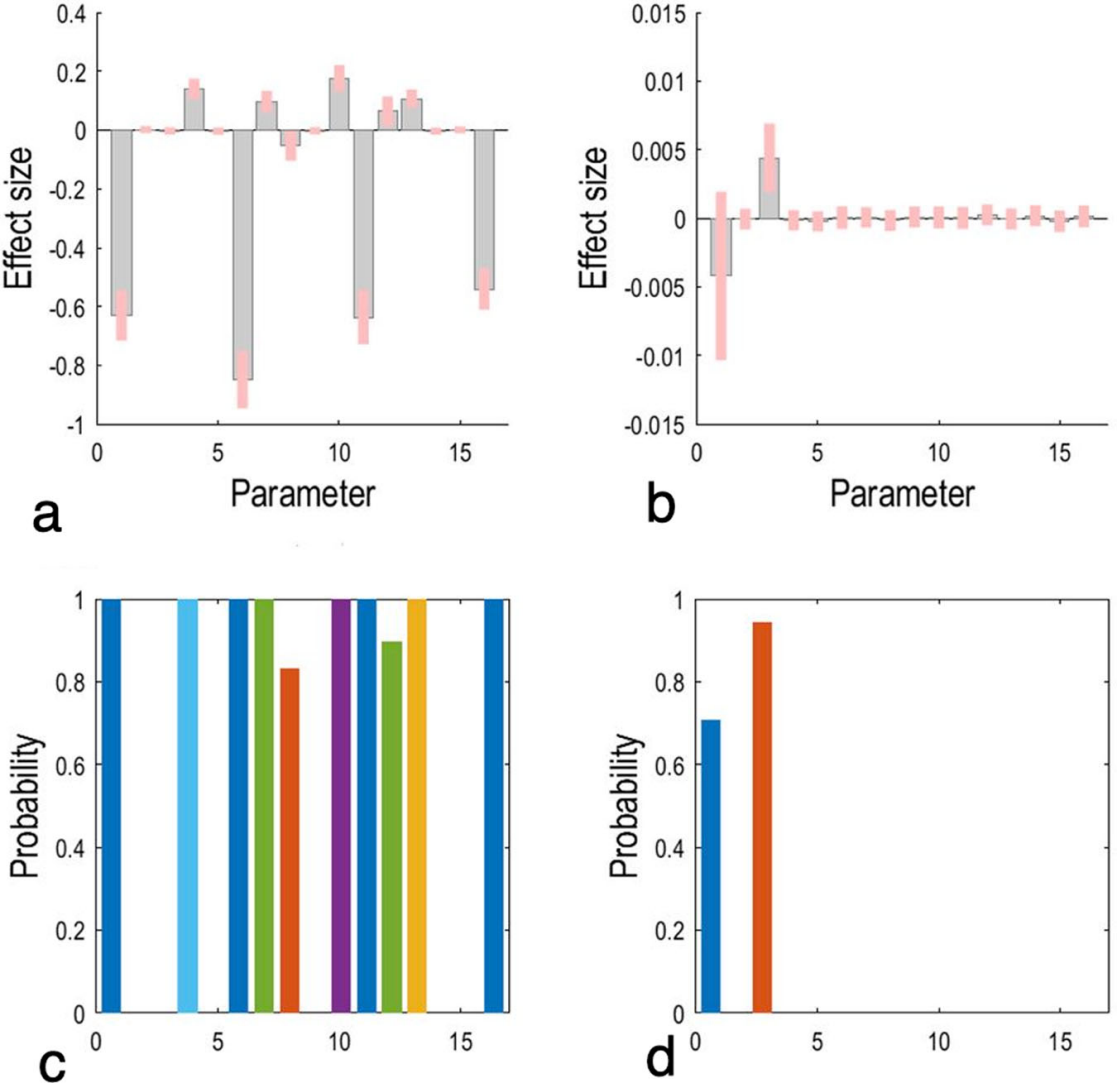
Correlation results between effective connections and IAT scores through parametric empirical Bayesian framework. **a** shows each parameter’s effect size after Bayesian model averaging for the mean of all subjects, and **c** shows the corresponding posterior probabilities. **b** shows the effect size of correlations between each parameter and IAT scores and **d** shows the corresponding posterior probabilities. Here the threshold of the posterior probability is set to 0.9, so the current results indicate that a positive correlation between connection strength and IAT is observed in effective connection from the MFG to left putamen (i.e., parameter 3 in **b**, **d**). IGD: 174 subjects; RGU: 244 subjects. Note: parameter 1: self-connection from left MFG to left MFG; parameter 2: effective connection from left MFG to left NAcc; parameter 3: effective connection from left MFG to left putamen; parameter 4: effective connection from left MFG to right MFG; parameter 5: effective connection from left NAcc to left MFG; parameter 6: self-connection from left NAcc to left NAcc; parameter 7: effective connection from left NAcc to left putamen; parameter 8: effective connection from left NAcc to right MFG; parameter 9: effective connection from left putamen to left MFG; parameter 10: effective connection from left putamen to left NAcc; parameter 11: self-connection from left putamen to left putamen; parameter 12: effective connection from left putamen to right MFG; parameter 13: effective connection from right MFG to left MFG; parameter 14: effective connection from right MFG to left NAcc; parameter 15: effective connection from right MFG to left putamen; parameter 16: self-connection from right MFG to right MFG; IGD: 22 subjects; RGU: 18 subjects.

Dynamic causal modelling results are shown in Fig. 3. For all subjects, the strength of effective connectivity from the left MFG to left putamen was positively correlated with their IAT scores (parameter 3, red bar in d). No significant correlation with IAT scores was observed in other connections (posterior probability < 0.9).

### Longitudinal data

**Fig. 4:**
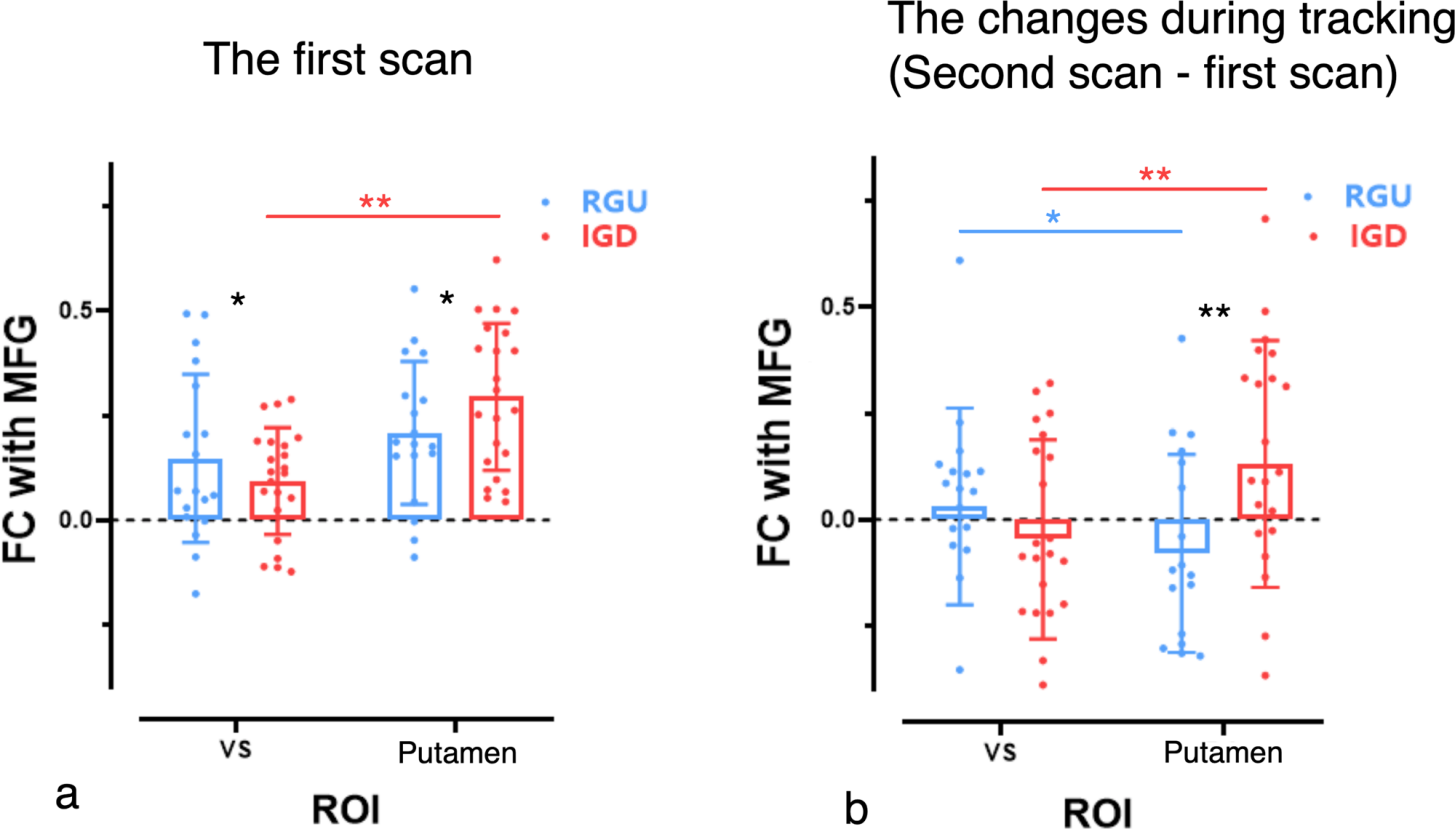
The VS-to-putamen involvement in longitudinal data. **a** The functional connectivity strength between ventral striatum/putamen and MFG in IGD and RGU groups in the first scan. **b** The functional connectivity strength changes between ventral striatum/putamen and MFG in IGD and RGU groups in the following scan relative to the first scan. *p < 0.05; **p < 0.01; ***p < 0.001.

In longitudinal data, group * VS/putamen interactions were observed in the first scan (*F*(1, 38) = 8.591, *p* = 0.006), the second scan (*F*(1, 38) = 16.191, *p* < 0.001), and second-minus-first scan (*F*(1, 38) = 10.47, *p* = 0.002) (Fig. 4). Further comparisons revealed that IGD subjects showed greater putamen-MFG functional connectivity than RGU subjects in the first scan (*t*(38) = 2.141, *p* = 0.024), the second scan (*t*(38) = 9.322, *p* < 0.001). When comparing the second scan minus the first scan, IGD relative to RGU subjects show a significant increase in the putamen-MFG functional connectivity (*t*(38) = 3.421, *p* = 0.007).

### False-positive assessment

We further tested whether global signal regression (GSR) influenced findings. With GSR, we observed similar results, especially in the left hemisphere (see Supplementary Figs. 1–3 and Supplementary Table 1). Together, the stability of the results in the left hemisphere suggest that the findings were not related to spurious noise^37^.

## Discussion

Using selected ROIs, the current study examined functional connectivity in the DS and VS in IGD and RGU subjects. We first identified an interactive effect between diagnostic group (IDG, RGU) and seed region (VS, DS) in functional connectivity with the MFG in a large sample. Next, we demonstrated similar results longitudinally in a sub-group of the subjects. Results are consonant in suggesting the relevance of DS versus VS connectivity to the MFG in IGD, raising the possibility of VS to DS shifts in IGD that have been proposed. The current results deepen our understanding of neural features underlying IGD and suggests potential common neural substrates between IGD and drug addictions.

### Ventral striatum and MFG

In the VS, IGD relative to RGU subjects showed lower functional connectivity with MFG, particularly on the left and mostly in the supplementary motor area (SMA). The SMA has been implicated in motor planning and execution, including self-initiated movements, action monitoring, response inhibition and action sequencing^38–41^. In fact, a significant relationship between SMA and striatum has been observed in people who use cocaine, and it was linked to poor processing of information^42^. The SMA may “link cognition to action”^43^ and act as a hub for processes related to motor intentionality^44,45^. The motor basal-ganglia-thalamocortical circuit model proposes that the circuit originates at the SMA, receiving input from primary and the premotor cortices^46^. These areas connect with the nuclei of the basal ganglia, which project back to the thalamus, closing the loop by reconnecting with the site of origin in motor cortical areas^46,47^. As the VS contributes to learning values of stimuli^5^, relatively decreased functional connectivity between the SMA and VS in IGD suggests a possible disconnection between stimulus evaluation and behavioural/motoric responses in domains including response inhibition.

In addition, a recent research also showed that resting activity of subcortical nucleus in obese subjects predicted their response to food-cues^48^. For the IGD, gaming cues that typically elicit higher cravings and reward-seeking in IGD versus RGU subjects^17,34^.

### Dorsal striatum and MFG

Longitudinal data indicated greater putamen-MFG functional connectivity in IGD relative to RGU subjects. Furthermore, IGD subjects showed a correlation between left putamen-MFG communication and DSM-5 scores that was absent in RGU subjects. The longitudinal data suggest that greater putamen-MFG functional connectivity persists over time. Greater functional connectivity between the DS and cortical regions has been linked to social anhedonia^49^ and negative mood states^50,51^, and greater functional connectivity between the DS and cortical regions have been implicated in major depression, although the findings vary across studies. As the current study excluded individuals with depression (or features thereof), additional research is needed to investigate whether putamen-MFG functional connectivity may be stronger in individuals with IGD and co-occurring depression. In addition, significant correlations between IGD severity and putamen-MFG functional connectivity suggest this relationship may underlie other clinically relevant aspects of IGD. Nonetheless, it should be noted that the effect size of this correlation is relatively small, so cautious interpretation is warranted. Together, the findings are consistent with theorical models of IGD^9,52,53^ that proposes that IGD is associated with neural adaptations in the striatum, including with DS networks as IGD linked to illness severity, similar to what has been proposed for another behavioural addiction, gambling disorder^54^.

Drug addictions have also been linked to neuroadaptations in the DS^4,5^. Thus, across behavioural and drug addictions, DS-cortical circuitry may relate importantly to habitual behaviours. The extent to which putamen-MFG functional connectivity relates to measures of habit and compulsivity in IGD warrants further direct examination.

The DCM findings further support the above results, suggesting that the greater the left putamen-to-MFG communication, the more severe the IGD. Generally, an imbalance within frontostriatal circuits is considered as a potential risk factor contributing to addiction. The dual-process theory of addictions proposes that a two-way relationship exists between prefrontal and striatal regions^55^. In this model, after an individual learns social rules, the prefrontal cortex controls the striatum via several mechanisms. However, this control is not absolute; hyperactivity within the striatum may override prefrontal control. The current results are consistent with top-down regulation of the prefrontal cortex on the striatal processing in IGD. Second, habit-oriented behaviours of addicted individuals have been proposed to be mediated by the functional activities involving the DS. Thus, we suggest that this top-down regulation may be linked to poor control in compulsive behaviours. Future studies should examine the extent to which possible shifts in VS-to-DS functional connectivity with the MFG relate to progression of more severe IGD, and greater DS-to-VS functional connectivity shifts with the MFG may relate to recovery.

### Limitations

Several limitations should be noted. First, more men than women were recruited in current study, which limits exploration of possible gender-related differences. Second, the study involved young adults from China and the extent to which the findings extend to other jurisdictions and cultures warrants additional investigation. Third, we used resting-state fMRI data in the current study, which may provide different results from task-based data. Task-based data (for example, of cue-elicited craving) may provide additional insight. Importantly, though, the large sample with resting-state fMRI is a strength with regard to rigour and reproducibility. Fourth, the current resting-state data acquisition were of 7 min duration, while recent research has demonstrated that longer scans may more reliably characterise individual differences in functional connectivity and brain network topology^56,57^. Thus, longer multi-echo fMRI sequences appear warranted in future resting-state studies. Fifth, this study used frame-to-frame head-motion estimators that may not perfectly correct for motion artefacts and run the risk of inflated false positives. An advanced denoising approach proposed by Ciric et al.^58^ may be a better choice. Sixth, the current study excluded subjects with depression and other psychiatric disorders. As such, the findings may not generalise to individuals with IGD and other co-occurring disorders. Future studies should examine individuals with IGD and co-occurring disorders like major depression. Seventh, in the current study, we used the number of proposed DSM-5 inclusion criteria that an IGD subject endorsed as a proxy measure of IGD severity. While the DSM-5 advocates such an approach for formal behavioural addictions like gambling disorder, future studies should consider additional measures of severity of IGD. Eighth, the current study suggested a neural transition feature in IGD, and such findings should be placed into an appropriate and not over-estimated position with respect to policymaking.

## Conclusions

In summary, the findings that IGD is linked to relatively greater putamen-MFG functional connectivity suggests a similar feature between more habitual and poorly controlled gaming in IGD and is consistent with proposed ventral-to-dorsal shifts in cortico-striatal circuitry involvement in addictions. Future studies investigating the malleability of the connectivity of such circuits to interventions are needed.

## Methods

### Ethics

The experiment conforms to the Code of Ethics of the World Medical Association (Declaration of Helsinki). The Human Investigations Committee of Hangzhou Normal University approved this research. All subjects were university students from Shanghai and were recruited through advertisements. All participants provided written informed consent before experimentation.

### Participant selection

Valid resting-state data from 174 IGD subjects and 244 RGU subjects scanned in 2016–2019 were included in the current study. Subjects were excluded from analyses for incomplete information or if their images had poor spatial normalisation, brain coverage by visual inspection, or excessive head motion (>3 mm and 3°).

Criteria for selection of IGD and RGU have been reported previously^34^ and are described briefly below. IGD status was determined based on scores of 50 or more on Young’s online internet addiction test (IAT, www.netaddiction.com)^59^ and concurrently meeting proposed DSM-5 criteria for IGD^60^. RGU participants were required to meet fewer than 5 (of 9) of the proposed DSM-5 criteria for IGD and score <50 on Young’s IAT.

All participants were right-handed and were university students recruited through advertisements. All participants provided written informed consent and underwent psychiatric interviews (using the Mini-International Neuropsychiatric Interview (MINI)^61^) performed by an experienced psychiatrist. Thus, IGD was diagnosed by a psychiatrist during a clinical interview. All participants were free of psychiatric disorders (including major depression, anxiety disorders, schizophrenia, and substance dependence disorders) as assessed by the MINI. Depression was further assessed with the Beck Depression Inventory (BDI) and those who scored >4 were excluded. Prior to fMRI, participants were asked to complete a 10-item gaming urge questionnaire that was based on a tobacco craving questionnaire, with each item using a 10-point response scale^62^. Thirty-one subjects were excluded from the current study, including 13 during pre-processing (poor spatial normalisation, considerable head movement), and 18 for discrepant categorisation between DSM-5 and IAT scores (Table 2).

**Table 2:** Demographics and clinical characteristics of all subjects.

	IGD	RGU		Group difference
	*N* = 174Male = 102Female = 72	*N* = 244Male = 149Female = 95	*t*	*p*-value
Demographics (M *±* *SD)*				
Age (year)	21.20 ± 2.441	21.56 ± 2.478	1.652	0.099
Years of education	14.57 ± 1.474	14.61 ± 1.402	−0.223	0.823
Clinical characteristics (M *±* *SD)*				
IAT	65.72 ± 8.783	40.09 ± 10.571	27.00	<0.001
DSM	6.12 ± 1.145	2.53 ± 1.430	27.093	<0.001
Gaming time (hours/week)	8.27 ± 3.616	6.17 ± 3.024	5.808	<0.001
Gaming history (year)	3.72 ± .640	3.73 ± .698	−0.130	0.894
Craving	51.79 ± 17.304	34.77 ± 16.280	9.253	<0.001

*p* < 0.05 was chosen as the significance level.

*IGD* Internet Gaming Disorder, *RGU* recreational game use, *M* mean, *SD* standard deviation, *IAT* Internet Addiction Test, *DSM* number of DSM-5 item.

### Measurement of gaming-craving

Prior to fMRI, participants were asked to complete a 10-item gaming urge questionnaire that was based on a tobacco craving questionnaire, with each item using a 10-point response scale{Cox, 2001 #58}. We modified the questionnaire according to the features gaming-related craving in IGD and tested the questionnaire in 275 subjects with a Cronbach’s alpha = 0.823 and split-half reliability = 0.803.

### Data acquisition

Resting-state functional data (T2*-weighted images) were acquired using a 3T Siemens Trio MRI scanner at the East China Normal University. Participants were instructed to keep their head still and eyes open during scanning. Earplugs and a head coil with foam pads were used to minimise machine noise and head motion. Specific parameters for structural scans were as follows. High-resolution T1-weighted three-dimensional spoiled gradient-recalled structural data were acquired for co-registration with the fMRI images (192 sagittal slices, TR = 2,530 ms, TE = 2.34 ms, inversion time = 1100 ms, FOV = 256 × 256 mm, flip angle = 78, matrix = 256 × 256, slice thickness = 1 mm, with a 50% gap). Specific parameters for resting-state scans are as follows: repetition time = 2000 ms, interleaved 33 axial slices, echo time = 30 ms, thickness = 3.0 mm, flip angle = 90°, field of view (FOV) = 220 mm × 220 mm, matrix = 64 × 64. Participants kept their eyes closed and were instructed not think of anything in particular during scanning. Each fMRI scan lasted 7 min, consisting of 210 imaging volumes. The resting-state fMRI data were accumulated over time from different studies, we used same parameters in each of the scans for resting state.

### Data pre-processing

Resting-state data analysis was performed using REST (figure view) and DPARSF (pre-processing, group comparisons) (http://restfmri.net)^63^. Pre-processing was performed using a standard approach that consisted of the following steps: (1) the initial 10 volumes were discarded, and slice-timing correction was performed; (2) the time series of images for each subject were realigned using a six-parameter (rigid body) linear transformation; (3) individual T1-weighted images were co-registered to the mean functional image using a 6 degrees-of-freedom linear transformation without re-sampling and then segmented into grey matter (GM), white matter (WM) and cerebrospinal fluid (CSF); (4) linear transformations with re-sampling to the voxel-sizes of [3 3 3] from individual native space to MNI space were computed with the DARTEL tool; (5) head-motion scrubbing using the Friston 24-parameter model to regress out head-motion effects; (6) mean framewise displacement (derived from Jenkinson’s relative root mean square algorithm) was used to address the residual effects of motion as a covariate in group analyses (other covariates included age and gender); and (7) further pre-processing included band-pass filtering between 0.01 and 0.08 Hz and smoothing with a 6-mm FWHM isotropic Gaussian kernel.

### Region of interest selection and seed-to-voxel functional connectivity

Anatomical seeds were chosen to increase the generalisability of findings^64,65^. We selected the bilateral NAcc (ventral striatum) and the bilateral putamen and caudate (dorsal striatum) based on the HarvardOxford-sub-maxprob-thr25 atlas (The putamen and caudate were considered as ROI separately).

For each seed, functional connectivity was acquired by Pearson correlation coefficients between the mean time series of the seed region and all brain voxels (defined by the binary GM mask in SPM). A Fisher’s Z transformation was applied to improve normality of correlation coefficient values. Finally, two-sample *t*-tests were applied to map group differences of connectivity maps for each seed between IGD and RGU participants.

### Group-level image processing

For group-level functional connectivity analyses, we performed two-sample *t*-tests to compare the connectivity maps of each seed between IGD and RGU participants. Corrections for multiple comparisons were conducted using permutation-based inferences (5000 permutations) with Threshold-Free Clustering Enhancement (TFCE), which provides strict control while improving replicability^66^. TFCE is a strict multiple-comparison correction strategy, which has been described as reaching the best balance between family-wise error rate (under 5%) and test–retest reliability/replicability relative to AFNI 3dClustSim, DPABI AlphaSim GRF, and false discovery rate^66^. For regions showing significant group differences (*t-*test), the mean Fisher Z-transformed correlation coefficients were extracted from the underlying anatomical regions for further post-hoc analysis. Group * VS/DS interaction was calculated and further post-hoc analyses were performed to explore the brain features. A *p* ≤ 0.05 threshold was considered as significant. These steps were performed by the pipeline software DPARSF (http://restfmri.net)^63^.

### Functional connectivity results with gaming history, symptom severity

We also correlated changes in functional connectivity with gaming history and addiction severity among IGD individuals. A *p* ≤ 0.05 threshold was considered as significant. The steps were performed with SPSS 20.0 (www.ibm.com).

### Spectral dynamic causal modelling

To provide insight into possible causal relationships between correlated brain regions, we performed spectral dynamic causal modelling (spDCM) analyses (spDCM is an extension of DCM under task-free state) (implemented by DCM12.5 (revision 7487), which is based on SPM12 (https://www.fil.ion.ucl.ac.uk/spm/)). A detailed description of the DCM specification can be found in our previous work (17). For each subject, the principal eigenvariate from the selected ROIs (i.e., the left VS (NAcc), the left DS (putamen) and the bilateral MFG) were first extracted and corrected for constructing subject-level DCM. Since we did not declare any prior assumptions about the directionality of the connections in the model, a single “full” model was then specified for each subject after extracting the principal eigenvariate. Here, the “full” model means that a two-way influence is assumed between the selected ROIs. Then, we completed the estimation of the subject-level DCM model by inverting the “full” models. Thus, there are no processes of model selection and comparison in the current study. Instead, we used an exploratory Bayesian model reduction in the group-level DCM analysis to automatically search over the optimal model from the ‘full’ model.

To effectively detect the relationship between addiction severity and effective connections, here we performed a Parametric Empirical Bayes (PEB) analysis to structure a hierarchical model over parameters. The PEB collates the subject-level DCM parameters and models at the group-level through a general linear model. It was enabled to capture any unexplained between-subject variability by the covariance component model. In the current study, the group-level PEB design matrix was coded as two-column regressors: (1) the commonalities across subjects (characterising by 1) and (2) the correlations between addiction severity and effective connections (characterising by IAT scores). An exploratory Bayesian model reduction was then performed to optimise the subject-level “full” model. Finally, A Bayesian model average was then calculated over the subject-level model models to obtain a group-level model. and the significance threshold of posterior probability in the model was set to 0.9.

### Replication test with longitudinal data

To investigate further, we tracked 40 subjects (22 IGD, 18 RGU) for more than 6 months and obtained additional data. These 40 subjects were part of the 418 subjects. We collected their resting-state data and addiction features during these two times of scanning. The details of these 40 subjects are listed in Supplementary Table 2. All pre-processing steps, group-level image processing and functional connectivity calculations were conducted similarly to those in the cross-sectional analyses. Group * VS/DS interactions were calculated in the first scan, the second scan and the second-first scan. A *p* ≤ 0.05 threshold was considered as significant. These steps were performed by the pipeline software DPARSF (http://restfmri.net)^63^.

### Statistics and reproducibility

All statistical steps were used open software and we did not do any modification on them. The parameters were provided on each of the statistical steps.

### Reporting summary

Further information on research design is available in the Nature Research Reporting Summary linked to this article.

## Supplementary information

Supplementary Information

Reporting Summary

## Data Availability

The data stored at our lab based network attachment system: http://QuickConnect.cn/others. ID:guests; PIN dong@123.COM.
