# Supplementary material for: Dorsal and ventral striatal functional connectivity shifts play a potential role in internet gaming disorder
Source: Commun Biol. 2021 Jul 14;4:866. doi: 10.1038/s42003-021-02395-5 (PMC8280218; doi:10.1038/s42003-021-02395-5)
Supplement: Supplementary file 1 — Supplementary Information [file 42003_2021_2395_MOESM1_ESM.pdf]

Supplementary materials for:

Dorsal and ventral striatal functional connectivity shifts play a potential role in internet gaming disorder

Guang-Heng Dong [1, 2,3*], Haohao Dong [4], Min Wang [1,2], Jialin Zhang [5], Weiran Zhou [2], Xiaoxia Du [6], Marc N.Potenza [,7,8,9]

[1] Center for Cognition and Brain Disorders, the Affiliated Hospital of Hangzhou Normal University, Hangzhou, P.R. China

[2] Institute of Psychological Research, Hangzhou Normal University, Hangzhou, P.R. China.

[3] Zhejiang Key Laboratory for Research in Assessment of Cognitive Impairments, Hangzhou, Zhejiang Province, P.R. China

[4] Department of Psychology, Zhejiang Normal University, Jinhua, P.R. China

[5] School of Psychology, Beijing Normal University, Beijing, P.R.China

[6] School of Psychology, Shanghai University of Sport, Shanghai, China

[7] Department of Psychiatry and Child Study Center, Yale University School of Medicine, New Haven, CT, USA

[8] Department of Neuroscience, Yale University, New Haven, CT, USA

[9] Connecticut Mental Health Center, New Haven, CT, USA

False-positive assessment

We assessed the authenticity of results by testing whether global signal regression (GSR) influenced findings. With GSR, we observed similar results, especially in the left hemisphere, similar conclusions can be drawn (See Supplementary Figure 1-3; Supplementary Table 1).

Supplementary Table 1: Results that show functional connectivity interactions between the VS and DS in the current study

cluster	Peak MNI coordinates			Peak Intensity	Cluster Size	Region	AAL
	X	Y	Z				
1	-9	-18	57	4.5541	24	L Middle Frontal Gyrus	Supp_Motor_Area_L
2	39	-12	57	4.0775	3	R Precentral Gyrus	Precentral_R

All images are thresholded at $p < .05$, TFCE-corrected; Iteration times =5000.
MNI: Montreal Neurological Institute; AAL: Anatomical Automatic Labelling;
TFCE:Threshold-Free Clustering Enhancement.

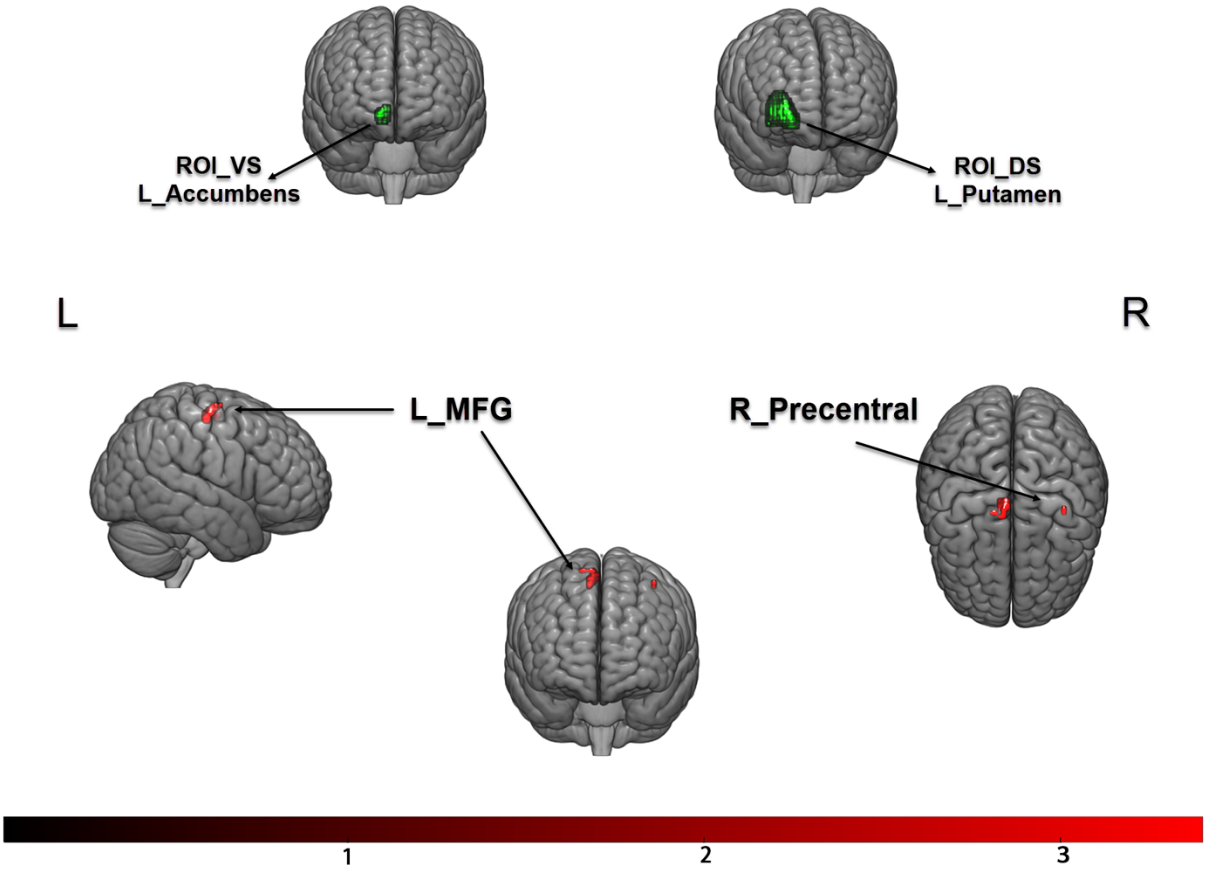

Supplementary Figure 1: Interactions were found between ventral/dorsal striatum and MFG

Upper: ROIs selected for ventral and dorsal in current study

Bottom: Brain regions that show FC interactions with left ventral/dorsal striatum

No significant results were observed in the right striatum.

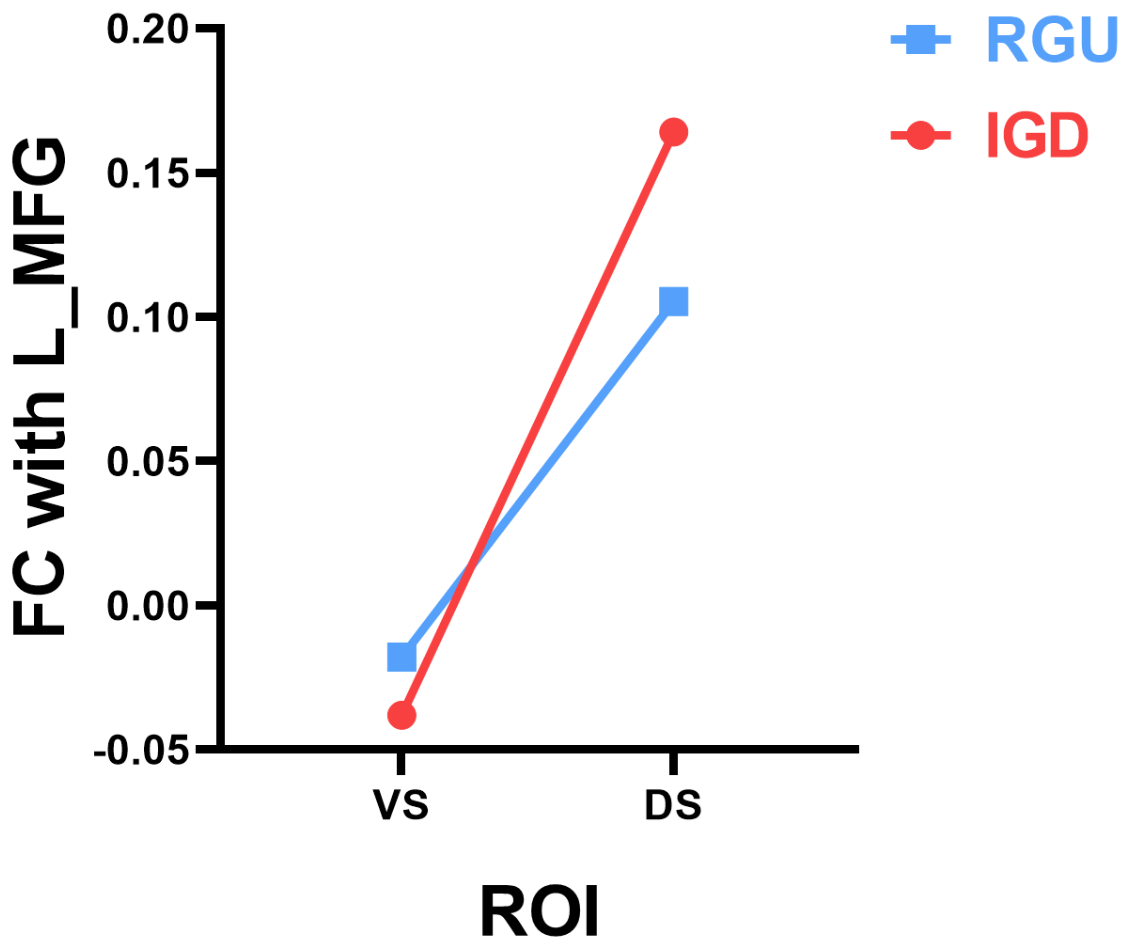

Supplementary Figure 2: The FC between ventral/dorsal striatum and MFG in different groups.

** $p<0.01$

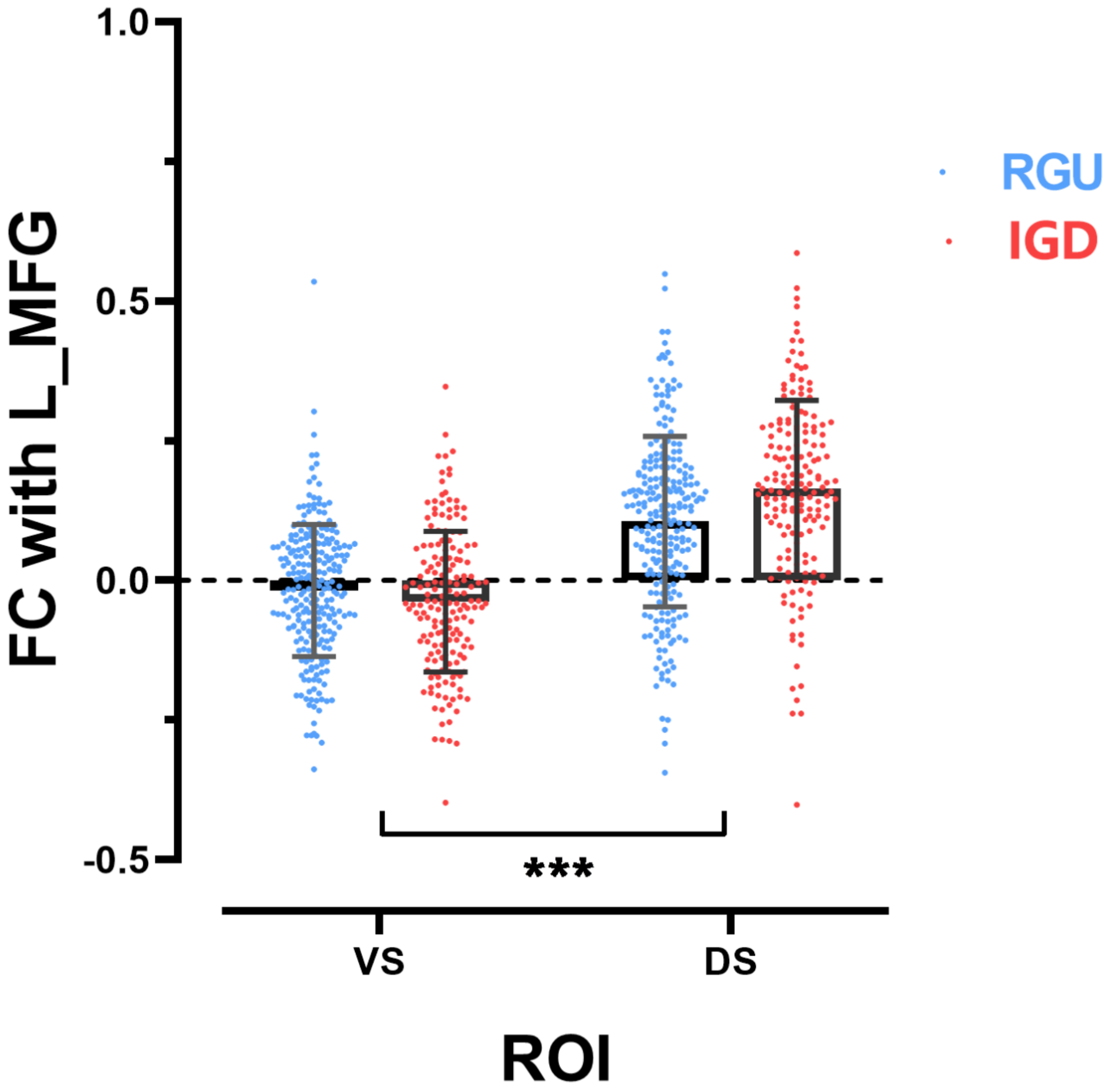

Supplementary Figure 3: Detailed features between VS/DS and MFG in different comparisons

The features in VS/DS FC with left MFG. * p<0.05; *** p<0.001

Supplementary Table 2. Demographic information of 40 tracked subjects

		IGD (n=22) 12 males	RGU (n=18) 9 males	*t*	*p*
Age (years)	pre-	*20.4±2.0*	*20.2±1.9*	*0.277*	*0.783*
	post-	*21.1±2.1*	*21.1±1.8*	*0.039*	*0.969*
Interval (months)		*9.2±3.5*	*9.3±3.6*	*-0.053*	*0.958*
IAT	pre-	*67.6±8.9*	*40.5±7.0*	*11.732*	*<0.001*
	post-	*68.7±7.9*	*36.7±8.0*	*11.402*	*<0.001*
DSM-5	pre-	*6.2±1.1*	*2.4±1.1*	*10.218*	*<0.001*
	post-	*6.5±1.3*	*2.3±1.4*	*9.180*	*<0.001*
Game time (hours per week)	pre-	*26.9±9.2*	*13.7±4.5*	*5.849*	*<0.001*
	post-	*27.6±10.3*	*17.0±8.4*	*3.500*	*0.001*
Craving	pre-	*53.0±19.3*	*31.7±16.3*	*3.711*	*0.001*
	post-	*54.9±14.7*	*33.8±14.5*	*4.528*	*<0.001*

IGD: Internet gaming disorder; RGU: recreational game user; IAT: Internet addiction test; DSM-5: Diagnostic and Statistical Manual of Mental Disorders-5; BDI: Beck Depression Inventory.
