# Supplementary material for: Dorsal and ventral striatal functional connectivity shifts play a potential role in internet gaming disorder
Source: Commun Biol. 2021 Jul 14;4:866. doi: 10.1038/s42003-021-02395-5 (PMC8280218; doi:10.1038/s42003-021-02395-5)
Supplement: Supplementary file 2 — Reporting Summary [file 42003_2021_2395_MOESM2_ESM.pdf]

nature research

Corresponding author(s): Guang-Heng Dong

Last updated by author(s): May 19, 2021

Reporting Summary

Nature Research wishes to improve the reproducibility of the work that we publish. This form provides structure for consistency and transparency in reporting. For further information on Nature Research policies, see our Editorial Policies and the Editorial Policy Checklist.

Statistics

For all statistical analyses, confirm that the following items are present in the figure legend, table legend, main text, or Methods section.

n/a	Confirmed	
☐	☒	The exact sample size (*n*) for each experimental group/condition, given as a discrete number and unit of measurement
☐	☒	A statement on whether measurements were taken from distinct samples or whether the same sample was measured repeatedly
☐	☒	The statistical test(s) used AND whether they are one- or two-sided<br>*Only common tests should be described solely by name; describe more complex techniques in the Methods section.*
☐	☒	A description of all covariates tested
☐	☒	A description of any assumptions or corrections, such as tests of normality and adjustment for multiple comparisons
☐	☒	A full description of the statistical parameters including central tendency (e.g. means) or other basic estimates (e.g. regression coefficient) AND variation (e.g. standard deviation) or associated estimates of uncertainty (e.g. confidence intervals)
☐	☒	For null hypothesis testing, the test statistic (e.g. F, t, r) with confidence intervals, effect sizes, degrees of freedom and P value noted<br>*Give P values as exact values whenever suitable.*
☒	☐	For Bayesian analysis, information on the choice of priors and Markov chain Monte Carlo settings
☒	☐	For hierarchical and complex designs, identification of the appropriate level for tests and full reporting of outcomes
☒	☐	Estimates of effect sizes (e.g. Cohen's *d*, Pearson's *r*), indicating how they were calculated

Our web collection on statistics for biologists contains articles on many of the points above.

Software and code

Policy information about availability of computer code

Data collection	Software with Siemens 3T scanner
Data analysis	REST,DPARSF

For manuscripts utilizing custom algorithms or software that are central to the research but not yet described in published literature, software must be made available to editors and reviewers. We strongly encourage code deposition in a community repository (e.g. GitHub). See the Nature Research guidelines for submitting code & software for further information.

Data

Policy information about availability of data

All manuscripts must include a data availability statement. This statement should provide the following information, where applicable:

- Accession codes, unique identifiers, or web links for publicly available datasets
- A list of figures that have associated raw data
- A description of any restrictions on data availability

All data are in the Network Attached Storage. People can reach it by the web link. To use them for publication must obtain author's permission.

Field-specific reporting

Behavioural & social sciences study design

All studies must disclose on these points even when the disclosure is negative.

Study description	Valid resting-state data from 174 IGD subjects and 244 RGU subjects scanned in 2016-2019 were included in the current study
Research sample	418
Sampling strategy	All participants were right-handed and were university students recruited through advertisements. All participants provided written informed consent and underwent structured psychiatric interviews (using the Mini-International Neuropsychiatric Interview (MINI)) performed by an experienced psychiatrist. All participants were free of psychiatric disorders (including major depression, anxiety disorders, schizophrenia, and substance dependence disorders) as assessed by the MINI. Depression was further accessed with Beck Depression Inventory (BDI) and those who scored higher than 4 were excluded. Prior to fMRI, participants were asked to complete a 10-item gaming urge questionnaire that was based on a tobacco craving questionnaire, with each item using a 10-point response scale.
Data collection	Criteria for selection of IGD and RGU have been reported previously and are described briefly below. IGD status was determined based on scores of 50 or more on Young's online internet addiction test (IAT, www.netaddiction.com) (33) and concurrently meeting proposed DSM-5 criteria for IGD . RGU participants were required to meet fewer than 5 (of 9) of the proposed DSM-5 criteria for IGD and score less than 50 on Young's IAT.
Timing	7 minutes
Data exclusions	Subjects were excluded from analyses for incomplete information or if their images had poor spatial normalization, brain coverage by visual inspection, or excessive head motion.
Non-participation	Cross sectional study, only valid participants included.
Randomization	Valid resting-state data from 174 IGD subjects and 244 RGU subjects

Reporting for specific materials, systems and methods

We require information from authors about some types of materials, experimental systems and methods used in many studies. Here, indicate whether each material, system or method listed is relevant to your study. If you are not sure if a list item applies to your research, read the appropriate section before selecting a response.

Materials & experimental systems

n/a	Involved in the study	
☒	☐	Antibodies
☒	☐	Eukaryotic cell lines
☒	☐	Palaeontology and archaeology
☒	☐	Animals and other organisms
☐	☒	Human research participants
☒	☐	Clinical data
☒	☐	Dual use research of concern

Methods

n/a	Involved in the study	
☒	☐	ChIP-seq
☒	☐	Flow cytometry
☐	☒	MRI-based neuroimaging

Human research participants

Policy information about studies involving human research participants

Population characteristics	see above
Recruitment	All participants were right-handed and were university students recruited through advertisements. All participants provided written informed consent and underwent structured psychiatric interviews (using the Mini-International Neuropsychiatric Interview (MINI))
Ethics oversight	The experiment conforms to the Code of Ethics of the World Medical Association (Declaration of Helsinki). The Human Investigations Committee of Hangzhou Normal University approved this research.

Note that full information on the approval of the study protocol must also be provided in the manuscript.

Magnetic resonance imaging

Experimental design

Design type	resting state

Design specifications	7 minutes continuous scan
Behavioral performance measures	No behavoral performance measures

Acquisition

Imaging type(s)	functional
Field strength	3T
Sequence & imaging parameters	EPI
Area of acquisition	whole brain

Diffusion MRI ☐ Used ☒ Not used

Preprocessing

Preprocessing software	DPARSF,(1) the initial 10 volumes were discarded, and slice-timing correction was performed; (2) the time series of images for each subject were realigned using a six-parameter (rigid body) linear transformation; (3) individual T1-weighted images were co-registered to the mean functional image using a 6 degrees-of-freedom linear transformation without re-sampling and then segmented into gray matter (GM), white matter (WM) and cerebrospinal fluid (CSF); (4) linear transformations with re-sampling to the voxel-sizes of [3 3 3] from individual native space to MNI space were computed with the DARTEL tool; (5) head-motion scrubbing using the Friston 24-parameter model to regress out head-motion effects; (6) mean framewise displacement (derived from Jenkinson's relative root mean square algorithm) was used to address the residual effects of motion as a covariate in group analyses (other covariates included age and gender); (7) further preprocessing included band-pass filtering between 0.01 and 0.08 Hz and smoothing with a 6mm FWHM isotropic Gaussian kernel.
Normalization	individual T1-weighted images were co-registered to the mean functional image using a 6 degrees-of-freedom linear transformation without re-sampling and then segmented into gray matter (GM), white matter (WM) and cerebrospinal fluid (CSF)
Normalization template	Firtst to T1, and then MNI template.
Noise and artifact removal	head-motion scrubbing using the Friston 24-parameter model to regress out head-motion effects; mean framewise displacement (derived from Jenkinson's relative root mean square algorithm) was used to address the residual effects of motion as a covariate in group analyses (other covariates included age and gender);
Volume censoring	DPARSF

Statistical modeling & inference

Model type and settings	resting state data
Effect(s) tested	We also correlated changes in functional connectivity with gaming history and addiction severity among IGD individuals. A P<0.05 threshold was considered as significant.

Specify type of analysis: ☐ Whole brain ☒ ROI-based ☐ Both

Anatomical location(s)	We selected the bilateral NAcc (ventral striatum) and the bilateral putamen and caudate (dorsal striatum) based on the HarvardOxford-sub-maxprob-thr25 atlas (The putamen and caudate were considered as ROI separately).
Statistic type for inference (See Eklund et al. 2016)	cluster wise
Correction	Corrections for multiple comparisons were conducted using permutation-based inferences (5,000 permutations) with Threshold-Free Clustering Enhancement (TFCE), which provides strict control while improving replicability

Models & analysis

n/a	Involved in the study
☐	☒ Functional and/or effective connectivity
☒	☐ Graph analysis
☒	☐ Multivariate modeling or predictive analysis

Functional and/or effective connectivity	We also correlated changes in functional connectivity with gaming history and addiction severity among IGD individuals. A P<0.05 threshold was considered as significant.
